# Altered polyunsaturated fatty acid levels in relation to proinflammatory cytokines, fatty acid desaturase genotype, and diet in bipolar disorder

**DOI:** 10.1038/s41398-019-0536-0

**Published:** 2019-08-27

**Authors:** Norie Koga, Jun Ogura, Fuyuko Yoshida, Kotaro Hattori, Hiroaki Hori, Emiko Aizawa, Ikki Ishida, Hiroshi Kunugi

**Affiliations:** 1 Department of Mental Disorder Research, National Institute of Neuroscience, National Center of Neurology and Psychiatry, Tokyo, Japan; 2 Department of Psychiatry, Yamanashi University Graduate School of Medicine, Yamanashi, Japan; 3 Department of Bioresources, Medical Genome Center, National Institute of Neuroscience, National Center of Neurology and Psychiatry, Tokyo, Japan; 4 Department of Behavioral Medicine, National Institute of Mental Health, National Center of Neurology and Psychiatry, Tokyo, Japan; 5 Department of Human Life Science, Nagoya University of Economics, Aichi, Japan

**Keywords:** Bipolar disorder, Molecular neuroscience

## Abstract

Inflammation and altered polyunsaturated fatty acid (PUFA) levels have been implicated in bipolar disorder (BD). A recent genome-wide association study identified a locus in the fatty acid desaturase (*FADS*) gene cluster conferring susceptibility to BD. In this study, we examined PUFA levels in patients with BD in relation to proinflammatory cytokines, *FADS* genotype, and dietary habits. We enrolled 83 patients with BD and 217 healthy controls who underwent plasma PUFA measurement. A subsample of 65 patients and 90 controls underwent plasma interleukin (IL)-6 and tumor necrosis factor alpha (TNFα) measurement, and three *FADS* single nucleotide polymorphisms (SNPs) were genotyped. Information on fish consumption was obtained by a self-reported diet history questionnaire. In comparing PUFA levels between patients and controls, significant differences were found for all 7 PUFAs tested. Specifically, n-3 eicosapentaenoic acid (EPA) level was decreased, and n-6 arachidonic acid level was increased in the patients (*p* < 0.0001 for both). Plasma IL-6 and TNFα levels were both significantly increased in the patients. Plasma EPA level was negatively correlated with IL-6 and TNFα levels. The *FADS* genotype, which was associated with increased n-6 PUFA levels, was also associated with marked elevation in TNFα levels. Less frequent fish intake was associated with low EPA and high IL-6 level. Taken together, our results provide strong evidence for altered plasma PUFA and proinflammatory cytokine levels in patients with BD. Furthermore, *FADS* genotype and fish consumption may contribute not only to altered PUFA levels but also to inflammation in BD.

## Introduction

Bipolar disorder (BD) is a recurrent chronic disorder characterized by fluctuations in mood and energy, affecting more than 1% of the world’s population^1^. It also affects patients’ daily life through cognitive and functional impairment and it increases the mortality of comorbid conditions such as cardiovascular diseases, diabetes mellitus, and suicide^2^. Current pharmacological treatment often lacks adequate efficacy in that residual mood symptoms often remain after initial treatment, and subsequent recurrence is frequent, which might be due to the lack of knowledge regarding the neurobiological mechanisms underlying the disorder^3,4^. An alternative approach is the use of dietary interventions, which seem to be promising^5,6^. Indeed, a substantial proportion of patients with BD are voluntarily using dietary supplements^7^.

Although the biological causes of BD have been elusive, abnormal monoamine functions, hypothalamus-pituitary-adrenal axis dysregulation, and chronic inflammation have been implicated^8–11^. Particularly, inflammation is thought to play a key role in the pathophysiology of psychiatric disorders^12,13^, including BD^14,15^. A recent meta-analysis of blood cytokine alterations showed increased proinflammatory cytokines such as interleukin (IL)-6 and tumor necrosis factor alpha (TNFα) in BD^16^.

Accumulating evidence suggests that polyunsaturated fatty acids (PUFAs) play a role in the inflammation of psychiatric patients. The group of n-3 (ω3) PUFAs can help treat inflammatory conditions, while arachidonic acid (AA), which belongs to n-6 (ω-6) PUFAs, facilitates inflammation^17–19^. The n-3 PUFAs have been suggested as an effective treatment of BD, although the results of randomized clinical trials were often negative^19–23^. Specifically, n-3 PUFAs may be effective for the treatment of bipolar depression, but not for mania^24^.

In contrast, there is a relative lack of evidence for altered PUFA levels in the blood of patients with BD. To our knowledge, seven research groups have reported plasma or red blood cell PUFA levels in BD patients compared with healthy controls^25–31^. A serious drawback was the small sample sizes; the first four studies enrolled only ≤20 BD patients, so it was difficult to draw any conclusions. Results of the subsequent three studies, in which the number of BD patients were somewhat higher (*N* = 27–42), were inconsistent. Evans et al.^29^ reported no significant difference in the plasma PUFA level between patients with BD and healthy controls. Pomponi et al.^30^ reported increased AA and EPA and decreased docosahexaenoic acid (DHA) in patients with BD. In contrast, Saunders et al.^31^ reported decreased EPA but no significant difference in AA or DHA in the patients. These inconsistent results require further investigation in a larger and more homogenous sample.

Interestingly, our recent genome-wide association study (GWAS) in Japanese patients with BD revealed a novel locus in the fatty acid desaturase gene cluster (*FADS1/2/3)*^32^. *FADS1* and *FADS2* are mainly expressed in the liver and catalyze the desaturation steps in the synthesis of n-3 and n-6 PUFAs. Polymorphisms in these genes have been shown to be associated strongly with variation in blood PUFA levels in humans^33,34^. This raises the intriguing possibility of examining the association between the genotype and inflammation.

It is well known that PUFA levels are greatly influenced by dietary habits as well. Fish consumption has been relatively high in Japan compared to European countries and the United States of America. For example, the per capita fish consumption of Japan in 2013 was 49.3 kg/year while those of European countries (28 countries) and the USA were 22.5 and 21.5 kg/year, respectively (data from the Food and Agriculture Organization of the United Nations). It is therefore intriguing to examine whether Japanese patients with BD show altered levels of PUFAs compared with controls. In addition, there is no study that has examined genetic and dietary factors simultaneously in relation to PUFAs and inflammation in BD.

The aims of the present study were fourfold. First, we examined whether plasma PUFA levels are altered in Japanese patients with BD when compared with healthy controls. Second, we examined proinflammatory cytokine levels of the patients and their correlation with PUFA levels. Third, we examined the association of *FADS* genotype with plasma PUFA and cytokine levels. Finally, we examined the relationship of dietary habits, particularly fish intake, with PUFA, cytokine levels, and the risk of BD.

## Materials and methods

### Subjects

Subjects for PUFA analysis were 83 patients with BD (20 bipolar I and 63 bipolar II) and 217 healthy volunteers (total: *N* = 300) recruited at the National Center of Neurology and Psychiatry (NCNP) hospital and the local community (Western Tokyo) through the NCNP website and local magazine advertisements. All participants were biologically unrelated Japanese who were interviewed for enrollment by using the Japanese version of the Mini-International Neuropsychiatric Interview^35,36^ and an additional unstructured interview. Diagnosis of BD was made by a board-certified psychiatrist according to the Diagnostic and Statistical Manual of Mental Disorders, 4th edition (DSM-IV)^37^, based on the information from the interviews and medical charts if available. The patients were rated on their manic and depressive symptoms using the Young Mania Rating Scale (YMRS)^38^ and the GRID Hamilton depression rating scale, 21-item version (HAMD21)^39,40^, respectively. The inter-rater reliability of our HAMD21 scoring was high (correlation coefficient *r* = 0.91)^41^. Healthy controls had no history of contact with any psychiatric services. Participants were excluded if they had a medical history of central nervous system diseases, severe head injury, substance abuse, or mental retardation. Patients were provided a written description of the study, and written informed consent was obtained from every participant. The study protocol was approved by the ethics committee at NCNP. The study was performed in accordance with the Declaration of Helsinki^42^.

### Measurement of plasma polyunsaturated fatty acid levels

After a fast of at least 5 h, venous blood was drawn between 11:00 and 14:00 to an ethylenediaminetetraacetic acid sodium-containing vacutainer tube (Terumo, Tokyo, Japan), and was immediately centrifuged at 3000 rpm for 15 min at 4 °C. Supernatant was collected into a polyethylene tube and stored at −20 °C. The sample was then delivered to SRL, Inc (Hachioji, Tokyo, Japan) where the concentrations of 7 different PUFAs (α-linolenic acid, EPA, and DHA for n-3 PUFAs; linoleic acid, γ-linolenic acid, dihomo-γ-linolenic acid, and AA for n-6 PUFAs) were measured using liquid chromatography/mass spectrometry.

### Cytokine assay

Among the total subjects whose PUFA levels were measured, a subsample of 65 patients with BD and 90 controls (see supplementary Table S1 for demographic and clinical data) underwent measurement of plasma IL-6 and TNFα. Plasma samples were obtained as described above, frozen in small aliquots, and stored at −80 °C until analysis. Investigators were blinded to the subjects’ clinical information, and IL-6 and TNFα measurement was performed using the MAGPIX CCD imaging system (MAGPIX xPONENT 4.2 system, Merck & Co., Inc.; Bio-Rad Laboratories, Inc.) using a magnetic on-bead antibody for each cytokine. Experiments were performed according to the manufacturer’s instructions.

### Single nucleotide polymorphism selection and genotyping

Genotyping was performed for the above subjects (65 patients with BD and 90 controls). Three single nucleotide polymorphisms (SNP), rs28456, rs174576, and rs174547, were selected based on previous studies^32,43^. The reasons for the SNP selection and genotyping methods are described in Supplementary information 1.

### Assessment of dietary intake

In the participants with plasma PUFA measurements, dietary intake was assessed using a brief self-administered diet history questionnaire^44,45^. From the questionnaire, we obtained information on frequency of fish intake in the preceding month. Frequency of 6 categories of fish consumption was assessed on the following 7-point scale: (1) no intake, (2) less than once a week, (3) once a week, (4) 2–3 times a week, (5) 4–6 times a week, (6) once a day, and (7) twice or more per day. Here we focused on fish intake because it is known to be associated with n-3 PUFAs such as EPA and DHA^46^.

### Statistical analysis

Methods of statistical analyses are described in Supplementary information 2.

## Results

### Characteristics of the subjects

Demographic and clinical characteristics of the subjects are shown in Table 1. BMI and the rate of smokers were significantly greater in patients than in controls. Characteristics of the patients concerning medication of mood stabilizers and depressive / manic episode are described in Supplementary information 3.

**Table 1:** Characteristics of patients with bipolar disorder and healthy controls for polyunsaturated fatty acid measurement

	Patients (*N* = 83)		Healthy controls (*N* = 217)		Statistical comparison
	Mean ± SD	Range	Mean ± SD	Range	
Age (years)	40.4 ± 9.9	18–67	41.6 ± 10.8	19–62	*t* = 0.87, df = 298, *P*= 0.39
Sex (No. of female, %)	46, 55.4%		136, 62.7%		*χ*^2^ = 1.32, df = 1, *P* = 0.25
Education (years)	15.4 ± 2.8	10–28	15.3 ± 2.4	10–26	*t* = −0.46, df = 298, *P* = 0.64
Body mass index (kg/m^2^)	24.3 ± 4.2	16.5–35.5	21.9 ± 3.4	16.1–32.8	*t* = −4.53, df = 124.0 ***P*** = **0.00001**
Smoker (*N*, %)	20, 24.1%		31, 14.3%		*χ*^2^ = 4.10, df = 1, ***P*** ***=*** **0.043**
Age at onset (years)	27.9 ± 10.5	13–57			
History of hospitalization (*N*, %)	28, 33.7%				
History of suicide attempt (N, %)	23, 27.7%				
^a^Antidepressants (*N* = 34)	118.9 ± 103.0	12.5–450			
^b^Antipsychotics (*N* = 13)	206.3 ± 161.3	37.9–500			
G-HAMD21	14.4 ± 7.1	1–33			
YMRS	2.1 ± 3.7	0–19			

*N* number, *SD* standard deviation, *df* degree of freedom, *G-HAMD21* Grid-Hamilton depression rating scale 21-item version, *YMRS* Young mania rating scale

^a^mean imipramine equivalent dose (mg/day) of antidepressants in patients with any antidepressant medication

^b^mean chlorpromazine equivalent dose (mg/day) of antipsychotics in patients under any antipsychotic medication

Significant *P*-values are indicated with bold cases

### Plasma PUFA levels

**Fig. 1:**
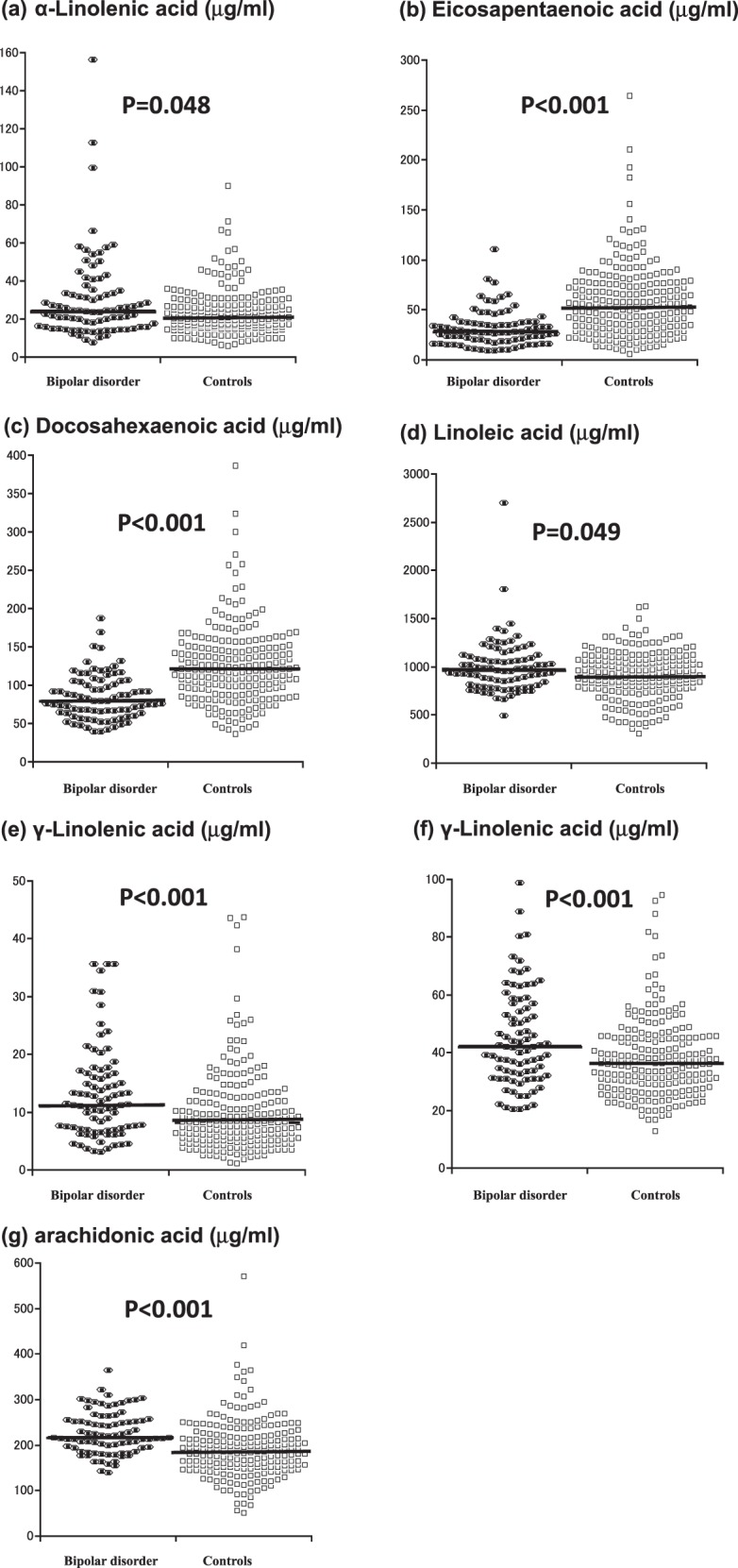
Comparison of plasma PUFA levels between the patients with bipolar disorder (*N* = 83) and healthy controls (*N* = 217). Horizontal bars indicate median values

Figure 1 and Table 2 show plasma PUFA levels in the patients and controls. The majority of distributions for PUFAs were deviated from the normal distribution (*P* < 0.05 by Shapiro-Wilk test) except for AA levels in patients and controls and linoleic acid level in controls. Plasma levels of all PUFAs were significantly different between the two groups (all *P* *<* 0.05 by Mann-Whitney *u* test). In particular, the differences in EPA, DHA, γ-linolenic acid, AA, and EPA/ AA ratio were highly significant (all *P* < 0.001). For the three n-3 PUFAs, α-linolenic acid level was increased, while levels of EPA and DHA were decreased in the patients relative to controls. In contrast, levels of the four n-6 PUFAs were all significantly increased in the patients relative to controls. As expected from these results, the EPA/AA ratio was significantly lower in the patients than in the controls. There was no significant correlation of the total HAMD21 score with any PUFA level (all *P* > 0.1 based on Spearman’s rank correlation test; data not shown).

**Table 2:** Plasma polyunsaturated fatty acid (PUFA) levels in patients with bipolar disorder and healthy controls

	Patients (*N* = 83)		Healthy controls (*N* = 217)		Statistical comparison^a^
	Median	1st to 3rd quartile	Median	1st to 3rd quartile	
n-3 PUFAs					
α-linolenic acid (μg/ml)	24.4	16.1–35.2	21.7	16.4–27.9	*U* = 1.98, ***P*** **=** **0.048**
eicosapentaenoic acid (EPA) (μg/ml)	27.9	18.7–37.8	54.2	31.4–76.8	*U* = −6.88, ***P*** **<** **0.001**
docosahexaenoic acid (μg/ml)	79.8	63.2–105.9	120.1	89.6–148.8	*U* = −7.19, ***P*** **<** **0.001**
n-6 PUFAs					
linoleic acid (μg/ml)	962.6	809.1–1110.2	909.8	779.3–1073.8	*U* = 1.97, ***P*** **=** **0.049**
γ-linolenic acid (μg/ml)	11.2	7.0–16.7	8.2	5.1–11.9	*U* = 3.86, ***P*** **<** **0.001**
dihomo-γ-linolenic acid (μg/ml)	42.0	32.0–57.0	36.5	28.5–45.2	*U* = 3.29, ***P*** **=** **0.001**
arachidonic acid (AA) (μg/ml)	217.3	194.3–255.2	188.4	152.0–223.9	*U* = 5.29, ***P*** **<** **0.001**
EPA/AA ratio	0.128	0.080–0.176	0.267	0.160–0.419	*U* = −8.26, ***P*** **<** **0.001**

*N* number, *SD* standard deviation

Significant *P* values are indicated with bold cases

^a^Mann–Whiteney *U* test; Standardized *U* values are shown

### Plasma cytokine levels and their correlation with PUFAs

**Fig. 2:**
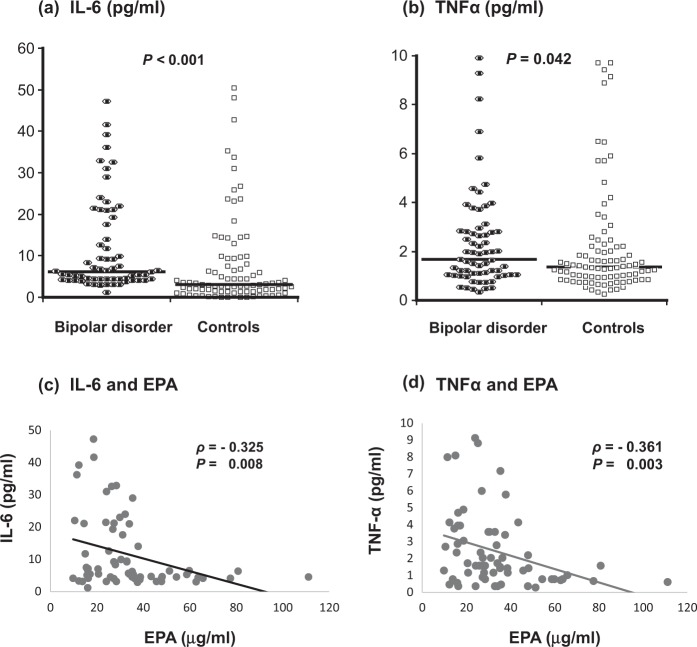
Plasma cytokine levels and their correlation with eicosapentaenoic acid level. **a** Comparison in interleukin (IL)-6 between the patients (*N* = 83) and controls (*N* = 217). **b** Comparison of tumor necrosis factor alpha (TNFα) between the patients and controls. **c** Correlation between plasma IL-6 and eicosapentaenoic acid (EPA) levels in the patients (*N* = 60). **d** Correlation between plasma TNFα and EPA levels in the patients

Demographic and clinical characteristics of the subjects who underwent cytokine measurement are shown in Supplementary Table S1. There was no significant difference in the age, sex ratio, education, or percentage of smokers between the patients and controls, while BMI was significantly greater in the patients. As shown in Fig. 2, cytokine distributions were skewed (all *P* < 0.001 by Shapiro-Wilk test); therefore, we used non-parametric tests in the following analyses. BMI had no significant correlation with cytokine levels in patients (Spearman’s *ρ* *=* −0.058, *P* *=* 0.65 for IL-6; *ρ* *=* 0.053, *P* *=* 0.67 for TNFα) or in controls (*ρ* *=* 0.177, *P* *=* 0.096 for IL-6; *ρ* *=* 0.121, *P* *=* 0.26 for TNFα). Both cytokine levels were significantly higher in patients than in controls (Fig. 2a, b, and Supplementary Table S2). Also, these levels were closely correlated in the patients (*ρ* *=* 0.45, *P* *=* 0.0002) as well as in controls (*ρ* *=* 0.32, *P* *=* 0.002). However, there was no significant correlation of the total HAMD21 score with either IL-6 or TNFα level (all *P* > 0.1 based on Spearman’s rank correlation test; data not shown).

Next, we examined the correlation between levels of PUFAs and cytokines for the patients and controls individually (supplementary Table S3). There were significantly negative correlations of EPA with IL-6 and TNFα levels in the patients (Fig. 2c, d). EPA/AA ratio negatively correlated with TNFα level in the patient group. In the controls, in contrast, there was a positive correlation between linoleic acid and TNFα levels.

### Association of FADS genotype with PUFAs and cytokines

Genotypes of the 3 SNPs were the same for all the genotyped subjects except for a single individual, indicating that the SNPs were in nearly absolute linkage disequilibrium. The genotype distributions and allele frequencies for rs174547 are shown in Supplementary Table S4. There were no significant differences in the genotype distribution or allele frequency between the patients and controls. When the association with plasma PUFA levels was examined, the genotype was significantly associated with plasma γ-linolenic acid and AA levels (both *P* < 0.001, by Kruskal–Wallis test) in the combined cohort (Supplementary Table S5). Even when the association was examined in the patients and controls separately, results were essentially similar for the two groups (data not shown). We then examined the possible association between the genotype and plasma cytokine levels (Supplementary Table S6). There were no significant differences in plasma IL-6 or TNFα level depending on the genotype in the combined cohort. Even when the association was examined for the patient and control groups individually, results were essentially unchanged (data not shown). Since abnormally high levels of cytokines may play a pathological role in inflammation, we further examined the possible association between genotype and an “abnormally high cytokine level.” Here we defined *a priori* an “abnormally high plasma cytokine level” as the upper 10^th^ percentile for all subjects (cut-off point for IL-6: 25.0 pg/mL; TNFα: 2.84 pg/mL). There was a significant linear-by-linear association between genotype and frequency of individuals with high TNFα in the combined cohort (*χ*^2^ = 6.0, df = 1, *P* = 0.014) where the C allele was associated with an abnormally high plasma TNFα level (Supplementary Table S7). However, such an association was not found between genotype and a high level of IL-6.

### Relationships of diet habits with PUFAs and cytokines

**Fig. 3:**
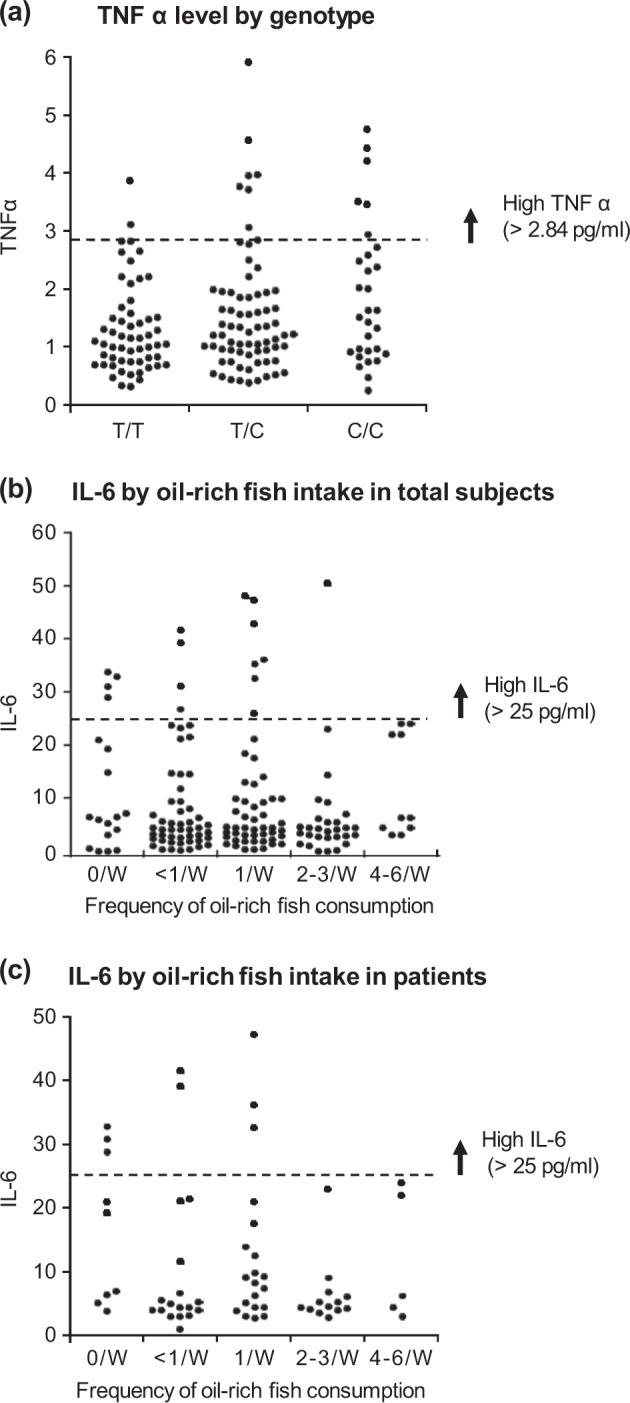
Distribution of proinflammatory cytokine levels depending on rs174547 of *FADS* genotype and frequency of oil-rich fish intake. **a** Plot of plasma tumor necrosis factor alpha (TNFα) level by rs174547 of *FADS* genotype in the total cohort (*N* = 155), indicating that the C allele is associated with greater frequency of elevated TNFα level (>2.84 pg/mL, indicated by the horizontal dotted bar). **b** Plot of plasma interleukin (IL)-6 level by oil-rich fish intake in the total cohort (*N* = 155), indicating that individuals who consumed oil-rich fish less frequently tended to be more likely to have high IL-6 level (>25.0 pg/mL, indicated by the horizontal dotted bar). **c**) Plot of IL-6 level by oil-rich fish intake in the patients (*N* = 65). There was no patient who had high IL-6 level among those who consumed oil-rich fish two or more times per week

Since fish intake increases EPA levels, and plasma EPA level was correlated with plasma cytokine levels in our patients (Fig. 2b, d), we focused on fish intake of the subjects. When correlation with plasma PUFA levels was examined, EPA best correlated with oil-rich fish consumption in the combined cohort (ρ = 0.26, *P* = 0.000007) (Supplementary Table S8). Oil-rich fish consumption was also correlated with plasma DHA level (ρ = 0.28, *P* = 0.000001). There was no significant correlation of oil-rich fish intake with plasma IL-6 (ρ = −0.089, *P* = 0.27) or TNF level (ρ = 0.021, *P* = 0.79). However, there was a linear-by-linear association between oil-rich fish and “high IL-6” (>25.0 pg/mL) with a trend toward statistical significance (*P* = 0.097) in the combined cohort. In patients with BD, the association reached statistical significance (*χ*^2^ = 4.7, df = 1, *P* = 0.029) (Fig. 3). However, there was no significant association of oil-rich fish intake with “high TNFα” (>2.84 pg/mL) (data not shown).

## Discussion

The data presented in this study lead to several key findings summarized here. First, we obtained strong evidence for altered PUFA levels in patients with BD compared with healthy controls. Plasma EPA and DHA levels (both n-3 PUFAs) were decreased, while AA level (n-6 PUFA) was increased in the patients. Second, plasma proinflammatory cytokine levels were increased in the patients compared with the controls. Further, EPA level significantly correlated with cytokine levels in the patients. Third, the *FADS* genotype was associated with differences in PUFA levels, particularly with levels of γ-linolenic acid and AA. Further, the *FADS* genotype was associated with high TNFα level. Finally, the frequency of oil-rich fish consumption, which correlated with plasma EPA level, was associated with “high IL-6”.

To our knowledge, this is the first study that examined PUFAs, proinflammatory cytokines, *FADS* genotype, and dietary fish intake simultaneously in BD patients and controls, which enables us to dissect the relationships across these pathological factors. Another advantage of the study is a relatively large sample size taken from a relatively homogeneous population (i.e., all subjects were Japanese).

We found that all 7 PUFA levels tested were significantly different between the patients and controls. Among n-3 PUFAs, EPA and DHA were decreased, and α-linolenic acid was increased in the patients. All n-6 PUFAs were increased in the patients. In particular, highly significant differences (*P* < 0.001) were seen for EPA, DHA, γ-linolenic acid, AA, and EPA/AA ratio. As mentioned above, previous studies on plasma PUFA levels have yielded mixed results. A small study (10 patients and 10 controls) by Sublette et al.^26^ found no significant differences in EPA, DHA, or AA. A larger study by Evans et al.^29^ investigated PUFA levels in 40 patients and 18 controls, but they also did not show any differences. Pomponi et al.^30^, in contrast, found increased EPA and AA and reduced DHA in a relatively large sample of 42 patients and 57 controls. Saunders et al.^31^ found reduced EPA in 27 patients compared to 31 controls. Our sample size is, to our knowledge, the largest yet reported (83 patients 217 controls), and yielded highly significant results. The reasons for the inconsistency are unclear; however, the relatively high rate of fish consumption by Japanese subjects as well as our large sample size may have contributed to the clear differences seen in our subjects. Given that n-3 PUFAs play a beneficial role in resolving inflammation and that n-6 PUFAs exert proinflammatory effects^17–19^, our findings fit well with the observation that at least a portion of patients with BD have increased levels of inflammation^14,15^. The finding of increased, rather than decreased, α-linolenic acid level in the patients was unexpected from this point of view and warrants further investigation. It is possible that conversion of α-linolenic acid to EPA is impaired in patients with BD.

In line with the observed decrease in EPA and DHA, which have been shown to resolve inflammation, the proinflammatory cytokines IL-6 and TNF-α were both found to be increased in patients with BD compared with healthy controls. These results are consistent with previous studies^14–16^. Although increased cytokine levels have been suggested to be correlated with severity^15^, we found no significant correlation between HAMD21 score and either cytokine level, which might have arisen from the heterogeneity in the etiology of the patients. When correlation with PUFA levels were examined, there were significantly negative correlations of EPA with IL-6 and TNFα levels in the patients. EPA/AA ratio also negatively correlated with TNFα level in the patient group. In the controls, there was a positive correlation between linoleic acid and TNFα levels. These results point to the importance of EPA among the PUFAs as a key player in inflammation of patients with BD. This finding is in accordance with the frequently replicated observation that EPA supplementation is more effective than DHA supplementation in treating or preventing psychiatric disorders^20,47,48^. In addition, we found a significant correlation between linoleic acid and TNFα levels in the controls, which is in line with the possible role of n-6 PUFAs in the causation of inflammation in BD^21^. Given that the use of dietary supplements including fish oil is common among patients with BD^7^, our results lend support for the use of supplementary EPA in the treatment of BD.

As expected, the *FADS* genotype was found to regulate PUFA levels. In our sample, the genotype for rs174547 correlated with n-6 PUFAs of γ-linolenic acid and AA. Although these PUFAs did not show significant correlation with either IL-6 or TNFα levels, another n-6 PUFA, linoleic acid, significantly correlated with TNFα level (supplementary Table S3). In line with this finding, the C allele, which corresponds to the risk allele for BD in our GWAS^32^, was found to be weakly associated with high TNFα level (the upper 10th percentile of all subjects). Although the risk-increasing effect of *FADS* genotype reported in our GWAS was weak (OR 1.18)^32^, our current study does provide a possible mechanistic explanation for how the genotype may contribute to the development of BD via increasing n-6 PUFA which leads to increased TNFα level and increased inflammation.

Finally, we examined the relationship of dietary fish intake with PUFA and cytokine levels. As expected, EPA and DHA levels showed a positive correlation with frequency of consumption of oil-rich fish (e.g., sardine, mackerel, saury, yellowtail) with a highly statistical significance (both *P* < 0.00001), suggesting the dietary information is reliable despite its self-reported nature. Although there was no association between PUFA and cytokine levels by the overall Kruskal–Wallis test, individuals who frequently consumed oil-rich fish were found to be less likely to show “high IL-6,” suggesting the possible role of dietary fish intake on the occurrence of inflammation. Our finding is in accordance with the study of Mocellin et al.^49^ who reported that n-3 PUFA supplementation reduced IL-6 level in people with cancer, although other studies have failed to find this association^50^.

Our study has several limitations. First, the severity of BD in our patients was relatively mild, and further studies are necessary to examine whether severe bipolar cases are related to more severe impairments in PUFA, cytokine, and imbalance in dietary habits. Second, the patients and controls differed in BMI, which may have contributed to the observed difference in cytokine levels. However, there was no significant correlation between BMI and cytokine levels, suggesting that the effect of BMI does not play a major role. Third, we examined only three SNPs of *FADS*, although there are other genes that regulate PUFA levels, such as elongase (*ELOVL2*) and glucokinase regulator (*GCKR*)^51,52^. Finally, the cross-sectional design of the present study makes it difficult to determine whether the observed relationships were causes or effects of the illness. Prospective studies with dietary and genetic information or clinical trials of dietary intervention are needed to elucidate the possible causal role of PUFA and cytokines in the pathogenesis of BD.

In conclusion, our results provide strong evidence for altered plasma PUFA levels and increased plasma proinflammatory cytokines in patients with BD. Reduced EPA levels are associated with increased proinflammatory cytokine levels. *FADS* genotype and fish consumption may contribute not only to altered PUFA levels but also to elevated cytokine levels in patients with BD.

## Supplementary information

Supplemental information 1 to 3

Supplementary Tables 1 to 8
