# Supplementary material for: Altered polyunsaturated fatty acid levels in relation to proinflammatory cytokines, fatty acid desaturase genotype, and diet in bipolar disorder
Source: Transl Psychiatry. 2019 Aug 27;9:208. doi: 10.1038/s41398-019-0536-0 (PMC6711984; doi:10.1038/s41398-019-0536-0)
Supplement: Supplementary file 1 — Supplemental information 1 to 3 [file 41398_2019_536_MOESM1_ESM.docx]

**Supplementary Information 1: Supplementary methods**

SNP selection

The reasons for the SNP selection were as follows: the SNP rs28456 (A to G transition; intron variant; effect allele on BD risk: G) located on *FADS2* encoding Δ-6 desaturase was the top hit (*P* = 6.4 × 10^−9^, odds ratio [OR] = 1.18) in our GWAS study on BD (ref. 32). The SNP rs174576 (C to A; intron variant; effect allele: A) located on *FADS2* was the top hit in the meta-analysis of the GWAS (*P* = 1.34 × 10^−10^, OR = 1.13). In addition, we selected the SNP rs174547 (T to C; intron variant; effect allele: C) because it is a well-established marker located in a haplotype block of *FADS1* encoding Δ-5 desaturase that regulate PUFA and levels (43). It also yielded a p-value of 1.3 × 10^−8^ (OR = 1.17) in the above-mentioned GWAS study.

Genotyping

Genomic DNA was prepared from venous blood according to standard procedures. We genotyped the 3 SNPs using the TaqMan 5-exonuclease allelic discrimination assay (assay ID: AHLJ253 for rs28456; C_2575520_10 for rs174576; C_2292336_10 for rs174547). The thermal cycling conditions for polymerase chain reaction were as follows: 1 cycle at 95° C for 10 min, followed by 50 cycles of 92° C for 15 s and 60° C for 1 min. The allele-specific fluorescence was measured with ABI PRISM7900 Sequence Detection Systems (Applied Biosystems, Foster City, CA). Ambiguous genotype data were not included in the analysis.

**References**

32) Ikeda M, Takahashi A, Kamatani Y, Okahisa Y, Kunugi H, Mori N, et al. A genome-wide association study identifies two novel susceptibility loci and trans population polygenicity associated with bipolar disorder. *Mol Psychiatry* 2018; **23(3)**: 639–647.

43) Guan W, Steffen BT, Lemaitre RN, Wu JH, Tanaka T, Manichaikul A, et al. Genome-wide association study of plasma N6 polyunsaturated fatty acids within the cohorts for heart and aging research in genomic epidemiology consortium. *Circ Cardiovasc Genet* 2014; **7**: 321–331.

**Supplementary Information 2: methods of statistical analysis**

The participants’ demographic and clinical characteristics are presented as mean ± standard deviation (SD) or the number of subjects. These variables were compared between the patients and controls by using a t-test for continuous variables and a χ^2^ test for independence for categorical variables. The Shapiro-Wilk test was used to examine the normality of distributions. Because plasma PUFA and cytokine levels were significantly deviated from normal distribution, non-parametric tests (Mann-Whitney test; Kruskal-Wallis test) were used to compare these values between the patients and controls. Spearman’s correlational analysis was also used. χ^2^ tests for goodness of fit, for independence, and for linear-by-linear association (Mantel-Haenszel test) were used to examine Hardy-Weinberg equilibrium of genotype distribution, comparison of frequency, and the trend in ordinal categories, respectively. Analyses were performed using the Statistical Package for the Social Sciences (SPSS) version 25.0 (Advanced Analytics Co., Ltd., Tokyo, Japan). Statistical significance was set at a two-tailed *P* < 0.05.

**Supplementary Information 3: clinical characteristics**

Mood stabilizer medication

Among the 83 patients, 5 patients were drug-free (untreated), and 45 were on at least one mood stabilizer (10 on lithium, 11 on valproate, 13 on lamotrigine, 1 on carbamazepine, and 10 on two or more of these drugs), and the remaining 33 were not prescribed any mood stabilizer.

Depressive / manic episode

The number of patients with depressed, manic, euthymic, or mixed episode at the time of study participation were 63, 1, 13, and 6, respectively according to the standard cut-off scores (HAMD21 score of 7 and YMRS score of 7). Thus, most subjects were depressed or euthymic, and there were only 7 patients whose YMRS score was 8 or greater.
