# Supplementary material for: Altered polyunsaturated fatty acid levels in relation to proinflammatory cytokines, fatty acid desaturase genotype, and diet in bipolar disorder
Source: Transl Psychiatry. 2019 Aug 27;9:208. doi: 10.1038/s41398-019-0536-0 (PMC6711984; doi:10.1038/s41398-019-0536-0)
Supplement: Supplementary file 2 — Supplementary Tables 1 to 8 [file 41398_2019_536_MOESM2_ESM.docx]

| **Supplementary Table S1 Characteristics of patients with bipolar disorder and healthy controls for cytokine measurement** | | | | | | | | | | | | | | | | |  |
| --- | --- | --- | --- | --- | --- | --- | --- | --- | --- | --- | --- | --- | --- | --- | --- | --- | --- |
|  | | | Patients (N=65) | | | | | |  | Healthy controls (N=90) | | | | | Statistical comparison | | |
|  | | | Mean ± SD / n, % | | | | | Range |  | Mean ± SD / n, % | | | | Range |  | | |
| Age (years) | | | 40.0 | | ± | 9.3 | | 22-64 |  | 40.0 | | ± | 11.7 | 20-62 | t=.011, df=151, *P*=0.99 | | |
| Sex (N of female, %) | | | 36 | |  | 55.4% | |  |  | 48 | |  | 53.3% |  | χ^2^=0.064, df=1, *P*=0.80 | | |
| Education (years) | | | 15.7 | | ± | 2.8 | | 12-22 |  | 15.4 | | ± | 3.2 | 10-22 | t=-0.449, df=153, *P* =0.65 | | |
| Body mass index (kg/m^2^) | | | 24.3 | | ± | 4.3 | | 16.5-33.1 |  | 22.5 | | ± | 3.7 | 16.1-32.5 | t=-2.87, df=153, ***P*=0.005** | | |
| Smoker (N, %) | | | 18 | |  | 27.7 % | |  |  | 16 | |  | 17.8% |  | χ^2^=2.167, df=1, *P*=0.14 | | |
| Age at onset (years) | | | 27.9 | | ± | 10.2 | | 13-49 |  |  | |  |  |  |  | | |
| History of hospitalization (N, %) | | | 20 | |  | 30.8% | |  |  |  | |  |  |  |  | | |
| History of suicide attempt (N, %) | | | 16 | | ± | 24.6% | |  |  |  | |  |  |  |  | | |
| ^†^Antidepressants (N=27) | | | 125.0 | | ± | 111.6 | | 12.5-450 |  |  | |  |  |  |  | | |
| ^‡^Antipsychotics (N=8) | | | 190.6 | | ± | 139.5 | | 75-450 |  |  | |  |  |  |  | | |
| G-HAMD21 | | | 14.4 | | ± | 7.7 | | 1-33 |  |  | |  |  |  |  | | |
| YMRS | | | 2.3 | | ± | 4.1 | | 0-19 |  |  | |  |  |  |  | | |
| † mean imipramine equivalent dose (mg/day) of antidepressants in patients with any antidepressant medication | | | | | | | | | | | | | | | | |  |
| ‡ mean chlorpromazine equivalent dose (mg/day) of antipsychotics in patients under any antipsychotic medication | | | | | | | | | | | | | | | | |  |
| N: number; SD: standard deviation; df: degree of freedom | | | | | | | | |  |  | |  |  |  |  | | |
| G-HAMD21: Grid-Hamilton depression rating scale 21-item version; YMRS: Young mania rating scale | | | | | | | | | | | | | | |  | |  |
| Significant *P* values are indicated in bold cases. | | | | | | | |  |  |  | |  |  |  |  | | |
| **Supprementary Table S2** Plasma cytokine levels (pg / ml) in patinets with bipolar diorder and healthy controls | | | | | | | | | | | | | | | |  |  |
|  |  |  | |  | | |  | | | |  | | | | |  |  |
|  | Patients with bipolar disorder | | | Healthy controls | | | | | | | Statistical comparison^$^ | | | | |  |  |
|  | Median | 1st to 3rd quartile | | Median | | | 1st to 3rd quartile | | | |  | | | | |  |  |
| IL-6 | 6.2 | 4.2 - 18.5 | | 3.1 | | | 1.4 - 9.4 | | | | U = 4.42, ***P* < 0.001** | | | | |  |  |
| TNFα | 1.37 | 0.96 - 2.57 | | 1.18 | | | 0.76 - 1.64 | | | | U = 2.03, ***P* = 0.042** | | | | |  |  |
|  |  |  | |  | | |  | | | |  | | | | |  |  |
| ^$^ Mann-Whiteney U test; Standardized U values are shown. | | | | | | |  | | | |  | | | | |  |  |
| IL-6: interleukin-6: TNFα: tumor necrosis factor α | | | | | | |  | | | |  | | | | |  |  |
| Significant *P* values are indicated in bold cases. | | | | | | |  | | | |  | | | | |  |  |

| **Supplementay Table S3** Correlation between plasma PUFA and cytokine levels in the patients and controls | | | | | | | | | |
| --- | --- | --- | --- | --- | --- | --- | --- | --- | --- |
|  |  | ALA | EPA | DHA | LA | GLA | DGLA | AA | EPA / AA |
| *Patients* |  |  |  |  |  |  |  |  |  |
| IL-6 | ρ | 0.244 | **-0.325** | -0.078 | 0.015 | -0.163 | 0.008 | -0.164 | -0.241 |
|  | *P* | 0.050 | **0.008** | 0.539 | 0.906 | 0.195 | 0.948 | 0.192 | 0.053 |
| TNFα | ρ | 0.182 | **-0.361** | -0.117 | 0.149 | -0.080 | 0.048 | -0.109 | **-0.315** |
|  | *P* | 0.147 | **0.003** | 0.352 | 0.238 | 0.529 | 0.703 | 0.387 | **0.011** |
|  |  |  |  |  |  |  |  |  |  |
| *Controls* |  |  |  |  |  |  |  |  |  |
| IL-6 | ρ | 0.113 | 0.126 | 0.138 | 0.200 | 0.044 | 0.093 | 0.071 | 0.126 |
|  | *P* | 0.287 | 0.237 | 0.196 | 0.059 | 0.681 | 0.382 | 0.505 | 0.236 |
| TNFα | ρ | 0.123 | 0.036 | 0.094 | **0.293** | -0.032 | 0.102 | 0.019 | 0.033 |
|  | *P* | 0.247 | 0.736 | 0.380 | **0.005** | 0.764 | 0.337 | 0.859 | 0.758 |
| Correlation was examined by Spearman's rank correlation test. | | | | | |  |  |  |  |
| Significant results (p<0.05) are indicated with red bold letters. | | | | | |  |  |  |  |
| IL-6: interleukin-6: TNFα: tumor necrosis factor α | | | | |  |  |  |  |  |
| ALA: α-linolenic acid; EPA: eicosapentaenoic acid; DHA: docosahexaenoic acid; LA: linoleic acid;  GLA: γ-linolenic acid; DGLA: dihomo-γ-linolenic acid; AA: arachidonic acid | | | | | | | | | |

| **Supplementary Table S4** Genotype distributions and alelle frequencies for rs174547 of *FADS* in the patients with bipolar disorder and controls | | | | | | | | |
| --- | --- | --- | --- | --- | --- | --- | --- | --- |
|  | | Genotype distribution | | | Total | allele frequency | |  |
|  |  | T/T | T/C | C/C |  | T | C | Total |
| Patients | Count | 24 | 33 | 8 | 65 | 81 | 49 | 130 |
|  | % | 36.9% | 50.8% | 12.3% | 100.0% | 62.30% | 37.70% | 100.0% |
| Controls | Count | 31 | 38 | 21 | 90 | 100 | 80 | 180 |
|  | % | 34.4% | 42.2% | 23.3% | 100.0% | 55.60% | 44.40% | 100.0% |
|  |  |  |  |  |  |  |  |  |
| The genotype distributions for the patients and controls were both in Hardy-Weinberg equilibrium (χ^2^ = 0.42, df=1, *P* = 0.51 for the patinets; χ^2^ = 1.9, df=1, *P* = 0.17 for the controls). There was no significant difference in the genotype distribution (χ^2^ = 3.1, df=2, *P* = 0.21) or allele frequency (χ^2^ = 1.4, df=1, *P* = 0.23) between the patients and controls. | | | | | | | | |

| **Supplementary Table S5** Association between rs174547 genotype and PUFA levels (μg/ml) in the total subjcts (N=155) | | | | | | | |
| --- | --- | --- | --- | --- | --- | --- | --- |
|  | rs174547 Genotype | | | | | |  |
|  | T/T (N=55) | | T/C (N=71) | | C/C (N=29) | | Comparison^$^ |
|  | Median | 1st to 3rd quartile | Median | 1st to 3rd quartile | Median | 1st to 3rd quartile |  |
| ALA | 24.5 | 17.5-33.7 | 23.5 | 17-28.8 | 20.7 | 14.6-29.3 | H = 1.5, *P* = 0.47 |
| EPA | 48.4 | 16.7-76.4 | 35.2 | 24-48.5 | 41.9 | 27.7-36.8 | H = 4.4, *P* = 0.11 |
| DHA | 100.2 | 82.3-147.8 | 90.5 | 71.7-116.3 | 98.3 | 71.6-119.7 | H = 2.0, *P* = 0.37 |
| LA | 1016.1 | 845.9-1123.9 | 953.3 | 799.2-1065.6 | 864.5 | 778-1048.1 | H = 2.4, *P* = 0.29 |
| GLA | 5.1 | 3.7-7.9 | 7.6 | 5.7-12.9 | 12.6 | 9.1-17 | H = 33.2, ***P* < 0.001** |
| DGLA | 32 | 27.2-48.8 | 38.4 | 29.8-47.4 | 39.6 | 31.3-48.5 | H = 1.6, *P* = 0.45 |
| AA | 159 | 144.6-194.3 | 203.3 | 166.5-225.7 | 219.1 | 181.9-254.1 | H = 19.1, ***P* < 0.001** |
| EPA / AA | 0.22 | 0.12-0.29 | 0.21 | 0.12-0.16 | 0.18 | 0.13-0.18 | H = 3.7, *P* = 0.15 |
|  |  |  |  |  |  |  |  |
| ^$^: Kruskal-Wallis test (df=2) | | | |  |  |  |  |
| ALA: α-linolenic acid; EPA: eicosapentaenoic acid; DHA: docosahexaenoic acid; LA: linoleic acid; GLA: γ-linolenic acid; DGLA: dihomo-γ-linolenic acid; AA: arachidonic acid | | | | | | | |
| Significant *P* values are indicated in bold cases. | | | |  |  |  |  |

| **Supplementary Table S6** Association between rs174547 genotype and plasma cytokine levels (pg/ml) in the total subjcts (N=155) | | | | | | | |
| --- | --- | --- | --- | --- | --- | --- | --- |
|  | rs174547 Genotype | | | | | |  |
|  | T/T (N=55) | | T/C (N=71) | | C/C (N=29) | | Comparison^$^ |
|  | Median | 1st to 3rd quartile | Median | 1st to 3rd quartile | Median | 1st to 3rd quartile |  |
| IL-6 | 4.5 | 1.9 - 9.2 | 4.6 | 2.9 - 13.0 | 3.6 | 2.0 - 11.7 | H = 1.5 , *P* = 0.47 |
| TNFα | 1.1 | 0.8 - 1.6 | 1.2 | 0.9 - 2.0 | 1.5 | 0.9 - 2.6 | H = 3.7, *P* = 0.15 |
|  |  |  |  |  |  |  |  |
| ^$^: Kruskal-Wallis test (df=2) | | | | | | | |
| IL-6: interleukin 6; TNFα: tumor necrosis fator α | | | |  |  |  |  |

| **Supplementary Table S7**  Frequencies of high cytokine levels by rs174547 genotype in the total subjects | | | | | | | |
| --- | --- | --- | --- | --- | --- | --- | --- |
|  | | rs174547 genotype | | | Total | Comparison | |
|  |  | T/T | T/C | C/C |  | high v. normal | |
| *IL-6* |  |  |  |  |  |  | |
| Normal IL-6 | Count | 51 | 63 | 25 | 130 | Pearson: χ^2^ = 1.0, df = 2, *P* = 0.61 | |
|  | % | 36.7% | 45.3% | 18.0% | 100.0% | Linear-by-Linear Association: | |
| High IL-6 | Count | 4 | 8 | 4 | 25 | χ^2^ = 0.97, df = 1, *P* =0.32 | |
|  | % | 25.0% | 50.0% | 25.0% | 100.0% |  | |
| *TNFα* |  |  |  |  |  |  | |
| Normal TNFα | Count | 53 | 63 | 23 | 130 | Pearson: χ^2^ = 6.1, df=2, ***P* = 0.048** | |
|  | % | 38.1% | 45.3% | 16.5% | 100.0% | Linear-by-Linear Association: | |
| High TNFα | Count | 2 | 8 | 6 | 25 | χ^2^ = 6.0, df=1, ***P* =0.014** | |
|  | % | 12.5% | 50.0% | 37.5% | 100.0% |  | |
| IL-6: interleukin 6; TNFα: tumor necrosis fator α; df: degree of freedom | | | | | | |  |
| High IL-6: > 25.0 pg/ml; high TNFα: > 2.84 pg/ml | | | | | | |  |
| Significant *P* values are indicated in bold cases. | | | | | | |  |

| **Supplementy Table S8** Correlation between plasma PUFA levels and fish intake frequency in the total subjects (N = 155) | | | | | | | |
| --- | --- | --- | --- | --- | --- | --- | --- |
|  | | Squid, shrimp, lobster, and shellfish | whole-eat fish | Canned tuna | Dried fish / salted fish | Oil-rich fish | Non-oil rich fish |
| ALA | ρ | 0.055 | -0.028 | 0.019 | 0.001 | 0.001 | 0.007 |
|  | p | 0.339 | 0.625 | 0.749 | 0.987 | 0.993 | 0.904 |
| EPA | ρ | 0.126^*^ | 0.190^**^ | 0.128^*^ | 0.128^*^ | 0.256^**^ | 0.146^*^ |
|  | p | 0.029 | 0.001 | 0.027 | 0.026 | 0.000007 | 0.011 |
| DHA | ρ | 0.207^**^ | 0.217^**^ | 0.018 | 0.158^**^ | 0.277^**^ | 0.187^**^ |
|  | p | 0.0003 | 0.00015 | 0.756 | 0.006 | 0.000001 | 0.001 |
| LA | ρ | -0.031 | -.159^**^ | -0.071 | -0.104 | 0.004 | -0.003 |
|  | p | 0.587 | 0.006 | 0.217 | 0.073 | 0.942 | 0.952 |
| GLA | ρ | -0.075 | -0.099 | -0.030 | -0.017 | -0.069 | -0.070 |
|  | p | 0.192 | 0.087 | 0.608 | 0.771 | 0.234 | 0.229 |
| DGLA | ρ | -0.060 | -0.167^**^ | -0.013 | -0.043 | -0.101 | -0.087 |
|  | p | 0.299 | 0.004 | 0.821 | 0.461 | 0.079 | 0.134 |
| AA | ρ | -0.020 | -0.104 | 0.022 | -0.023 | 0.030 | -0.029 |
|  | p | 0.734 | 0.072 | 0.708 | 0.697 | 0.609 | 0.617 |
| *: P < 0.05, **: P < 0.01 based on Sperman's rank correlation test | | | | |  |  |  |
| ALA: α-linolenic acid; EPA: eicosapentaenoic acid; DHA: docosahexaenoic acid; LA: linoleic acid; GLA: γ-linolenic acid; DGLA: dihomo-γ-linolenic acid; AA: arachidonic acid | | | | | | | |
